# Evaluating the Impact of Clinical Mastitis on Ovarian Morphometry and the Effectiveness of the Synchronisation Protocol in Dairy Cows

**DOI:** 10.3390/ani15152215

**Published:** 2025-07-28

**Authors:** Gabija Lembovičiūtė, Greta Šertvytytė, Ramūnas Antanaitis, Vytuolis Žilaitis, Walter Baumgartner, Arūnas Rutkauskas

**Affiliations:** 1 Large Animal Clinic, Veterinary Academy, Lithuanian University of Health Sciences, Tilžės Str. 18, LT-47181 Kaunas, Lithuania; greta.sertvytyte@lsmu.lt (G.Š.); ramunas.antanaitis@lsmu.lt (R.A.); vytuolis.zilaitis@lsmu.lt (V.Ž.); arunas.rutkauskas@lsmu.lt (A.R.); 2 Clinical Centre for Ruminant and Camelid Medicine, University of Veterinary Medicine, Veterinaerplatz 1, A-1210 Vienna, Austria; walter.baumgartner@vetmeduni.ac.at

**Keywords:** oestrus, mastitis, synchronisation protocol

## Abstract

Inflammatory processes within the body have been hypothesised to be causative agents of various health complications, including reproductive issues. This study investigates the effects of mastitis on ovarian morphometry and fertility outcomes while also comparing the effectiveness of different synchronisation protocols in affected cows. Healthy multiparous Holstein dairy cows, in their second to fifth lactations and with an average milk yield of approximately 12,000 kg in the preceding lactation, were selected for this study. The average milk yield per lactation was approximately 12,000 kg, with an average daily milk yield of 30 kg. The aim of this study was to determine the impact of mastitis on the reproductive cycle, ovarian morphometry, and function in dairy cows, as well as its effect on synchronisation efficiency. This study focused on both clinically healthy cows and those diagnosed with clinical mastitis postpartum. Three different synchronisation protocols—OvSynch, G7G, and Presynch—were evaluated at 60 days in milk (DIM). A total of 110 cows were included in this study, with 53 being clinically healthy and 57 affected by mastitis. The results indicated that inflammation affects ovarian morphometric parameters such as the area and the number of functional structures. In addition, the G7G protocol was observed to result in higher conception rates in cows suffering from mastitis following the second insemination. This study demonstrated that cows that developed mastitis within the first 30 DIM exhibited higher conception rates after the first insemination compared to those in cows that developed mastitis later in lactation.

## Simple Summary

The overall population of cattle is in decline, whilst the size of agricultural enterprises is increasing; nevertheless, there is indication of an improvement in the overall productivity of farming. Reproductive losses are caused by several factors, with mastitis being particularly impactful. Mastitis is one of the most common and costly diseases in dairy cattle, affecting milk yield, reproductive performance, and profitability. Understanding the link between mastitis and reproductive performance is crucial to preventing its negative effects on fertility. Morphometric data on the ovaries are useful for evaluating and predicting the reproductive status of cows, as well as for selecting the appropriate synchronisation protocols. It is critical to investigate the interplay between mastitis, ovarian morphology, and reproductive management strategies in order to enhance fertility and overall productivity in the dairy sector. This study hypothesises that the presence of clinical mastitis in early lactation alters ovarian morphology and impairs reproductive outcomes and that the effectiveness of synchronisation protocols varies depending on the timing and severity of the mastitis. The significance of this topic is evidenced by its vital role in optimising the reproductive performance of dairy farms, a factor that is crucial for enhancing overall productivity and profitability. This is particularly pertinent in the context of diseases such as mastitis, which present not only challenges to the health of the cow but also to farm economics.

## 1. Introduction

The reproductive outcomes of dairy cows are influenced by a number of factors, including the cows’ health during the transition period, metabolic and udder health, hoof condition, the accurate detection of oestrus, and the conditions for semen preparation [1]. Inflammatory diseases have been observed to negatively impact the reproductive system by disrupting the transmission of endocrine signals, follicular development, and ovarian function [2,3]. It is also well documented that cows which experience diseases during early lactation often go on to have prolonged anovulatory periods [3,4]. Furthermore, research indicates that predominant pathogens impact the uterine microflora, leading to impaired embryo implantation and foetal development, which results in a higher incidence of abortions. The impact of mastitis on fertility is characterised by alterations in the oestrous interval and the luteal phase, which can disrupt the process of conception and embryo development [3,4].

It is crucial to recognise the elevated risk of disease in dairy cows during the postpartum period, which is characterised by significant physiological, endocrine, and metabolic changes within the body. Epidemiological studies have demonstrated that 40–60% of dairy cows undergo one or more clinical disease episodes within the 30–60 days following parturition [5]. This heightened susceptibility to diseases can be attributed to immune suppression resulting from a negative energy balance (NEB) during the initial phase of lactation, characterised by a reduced dry matter intake (DMI). A reduced DMI is a risk factor for postpartum disorders, such as postpartum paresis, metritis, and abomasal displacement, as well as associated reductions in milk production [6]. Furthermore, as DMI remains reduced during this time, the energy demand for milk production is insufficient. The presence of pathogens in the environment, spread within the housing areas, constitutes an additional risk factor for the health of dairy cows [7]. Feeding-related factors that can be linked to reduced mammary gland defence mechanisms include feed quality, feeding practices, changes in ration, and adjustments to individual feeding strategies [8]. A decline in average DMI as a percentage of body weight has been demonstrated to result in an elevated risk of ketosis and clinical mastitis [9]. Brennecke et al. [10] reported that greater variation in dry matter intake is associated with a higher occurrence of clinical mastitis due to environmental pathogens.

Bovine mastitis is defined as an inflammatory response in the mammary gland’s parenchymal tissue, resulting from physical injury or infection caused by microorganisms. The consequences of mastitis extend beyond the mammary gland itself, resulting in a systemic immune response and endocrine changes within the body. The aetiology of the disease is diverse, with various bacteria, mycoplasmas, and viruses being causative agents. Bacterial mastitis is the most common form of the disease and has the most significant physiological and economic impact. The causative agents of mastitis include both Gram-positive and Gram-negative bacteria. Gram-positive bacteria include various species of staphylococci and streptococci, while the most common Gram-negative bacteria are *Escherichia coli* and *Klebsiella pneumoniae* [11,12]. The endotoxin known as lipopolysaccharide (LPS) acts as the primary component of the cellular membrane of Gram-negative bacteria. It has been established that LPS can provoke a local or systemic inflammatory response through the stimulation of the innate immune system [13]. LPS stimulates cellular immune responses and cytokine release, thereby affecting the hypothalamic–pituitary–gonadal axis, resulting in increased follicular atresia, delayed oestrus, and failure to reach calving [12].

### Hormonal Regulation of Ooestrus Synchronisation in Cows

A considerable number of dairy farms worldwide have employed the strategy of timed artificial insemination (AI) with a view to achieving the optimal productivity and enhancing reproductive performance [14]. This approach has been shown to reduce the incidence of sexually transmitted diseases and enhance productivity by utilising semen from genetically superior bulls [15]. For the past seven decades, reproductive physiologists have been engaged in the development of programmes designed to synchronise oestrus and ensure high fertility. The success of these programmes has led to a growth in the interest in and understanding of female reproductive characteristics, including ovarian function and hormonal regulation of the bovine oestrus cycle. The application of ultrasonography and the development of more precise hormonal analysis techniques have enhanced our understanding of bovine reproductive physiology, particularly with a focus on ovarian function [15,16]. The oestrous cycle is a physiological phenomenon that can be subject to pharmacological regulation by hormonal treatments, which have been shown to effectively control the timing of ovulation [17]. The objective of synchronisation is to manage the onset of oestrus by regulating the duration of the reproductive cycle. The methods employed for the regulation of cycle length include the following: The first method involves regressing the corpus luteum (CL) to induce natural luteolysis, thereby shortening the cycle through the administration of prostaglandin F2α (PGF2α). A second method involves the administration of exogenous progestins to delay the onset of oestrus after natural or induced luteolysis, which may extend the duration of the reproductive cycle [12]. It can be hypothesised that the efficacy of these synchronisation protocols is intrinsically dependent upon the underlying ovarian physiology, which can be quantitatively and qualitatively characterised, at least in part, using specific morphometric parameters.

**Figure 1:**
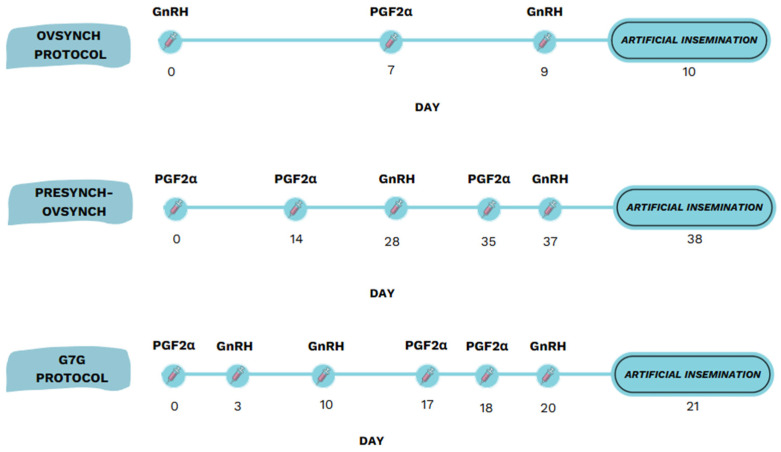
Timelines of OvSynch, Presynch–OvSynch, and G7G protocols. GnRH: gonadotropin-releasing hormone; PGF2α: prostaglandin F2α. The aim of this study was to evaluate the impact of clinical mastitis on ovarian morphometry and related reproductive outcomes in dairy cows, as well as to assess the effectiveness of different oestrous synchronisation protocols in cows with and without clinical mastitis. The effects were assessed by comparing ultrasonographically measured ovarian parameters (area, number, and size of follicles and corpora lutea) and fertilisation efficiency following synchronisation and artificial insemination between healthy cows and those diagnosed with clinical mastitis.

A range of hormonal synchronisation protocols, such as OvSynch, Presynch–OvSynch, and G7G–OvSynch, have been developed to optimise the timing of ovulation and improve fertility outcomes in dairy cows (Figure 1). The OvSynch protocol, combining gonadotropin-releasing hormone (GnRH) and prostaglandin F2α (PGF2α), is a widely used method to synchronise ovulation in dairy cows (Figure 1) [16]. When initiated between days 5 and 12 of the oestrous cycle, it improves the synchronisation outcomes by aligning follicular development [17]. Cows unresponsive to the first GnRH injection may develop persistent follicles, negatively affecting oocyte quality and early embryo development. Lavon et al. [18] reported that applying OvSynch in cows with subclinical mastitis improved their conception rates (40.5%) to levels comparable with those in healthy cows, suggesting its potential to mitigate mastitis-related reproductive delays. The Presynch–OvSynch (PO) protocol incorporates two prostaglandin F2α (PGF2α) injections 14 days apart prior to initiating the OvSynch sequence. This presynchronisation improves fertility outcomes by increasing the proportion of cows in the optimal stage of the oestrous cycle and enhancing their ovulatory response to GnRH [19]. The 14-day interval between the second PGF2α dose and the first GnRH injection is widely adopted on dairy farms to improve timing efficiency and simplify management [20]. The G7G–OvSynch protocol is a modified version of OvSynch, incorporating a GnRH injection and a PGF2α treatment seven days apart prior to the OvSynch sequence. This 20-day protocol improves ovarian follicular dynamics, resulting in a higher ovulation rate after the first GnRH injection and increased conception rates compared to these values under standard OvSynch. Studies have reported ovulation in up to 85% of cows when the protocol begins on day 6 of the oestrous cycle [21,22].

## 2. Materials and Methods

### 2.1. Ethical Approval

The present study was conducted in accordance with the regulations established by the Lithuanian Law on Animal Welfare and Protection and received approval under reference number G2-227 (dated 7 March 2025).

### 2.2. Keeping and Feeding of the Experimental Cows

The experiment was carried out on a commercial dairy farm with approximately 1500 milking cows in central Lithuania (54.9738 N, 23.7695 E) between August and November 2024. All cows that became eligible for synchronisation 60 days from parturition during that period were enrolled, yielding a convenience sample of *n* = 110. Table 1 presents the nutritional composition of the diet. Consequently, no a priori power calculation was undertaken. Nonetheless, this sample size proved sufficient to detect statistically significant differences between groups in key outcomes such as ovarian dimensions and synchronisation response. In the context of previous research, the utilisation of comparable group sizes has yielded favourable outcomes. For instance, Kavousi Nodar et al. [23] divided 110 Holstein cows into two equal groups (55 + 55) when assessing eCG presynchronisation prior to OvSynch.

### 2.3. Creation of the Experimental Groups

The investigation was conceived and conducted as a prospective observational study. Its primary objective was to evaluate the ovarian morphometry, reproductive performance, and inflammatory status of lactating dairy cows afflicted by clinical mastitis. A total of 110 postpartum cows were selected to monitor their health status concerning mastitis from the first day postpartum (pp). The cows under investigation were Holstein Friesians in their second to fifth lactation. The average milk yield per lactation was approximately 12,000 kg, with an average daily milk yield of 30 kg. The cows selected for this study had uncomplicated calving.

A total of 110 cows without dystocia or postpartum disorders, except for clinical mastitis, were selected for this study. Subclinical mastitis cases without visible milk abnormalities and not confirmed by somatic cell count (SCC) testing were not included. The diagnosis of clinical mastitis was based on the presence of visible abnormalities in the milk, including a watery consistency and the presence of clots. No bacterial culture or pathogen typing was performed; therefore, the etiological agent (i.e., bacteria, virus, fungi, or algae) was not identified. In this study, the inflammatory status of dairy cows was evaluated based on a clinical diagnosis of mastitis, which was identified by the presence of visible abnormalities in the milk. This practical approach aligns with the standard on-farm diagnostic methods and serves as an indicator of local inflammation in the mammary glands. Despite the absence of specific inflammatory biomarkers, the presence of clinical signs was deemed adequate to reflect an active inflammatory state that was meaningful to the study’s objectives.

**Figure 2:**
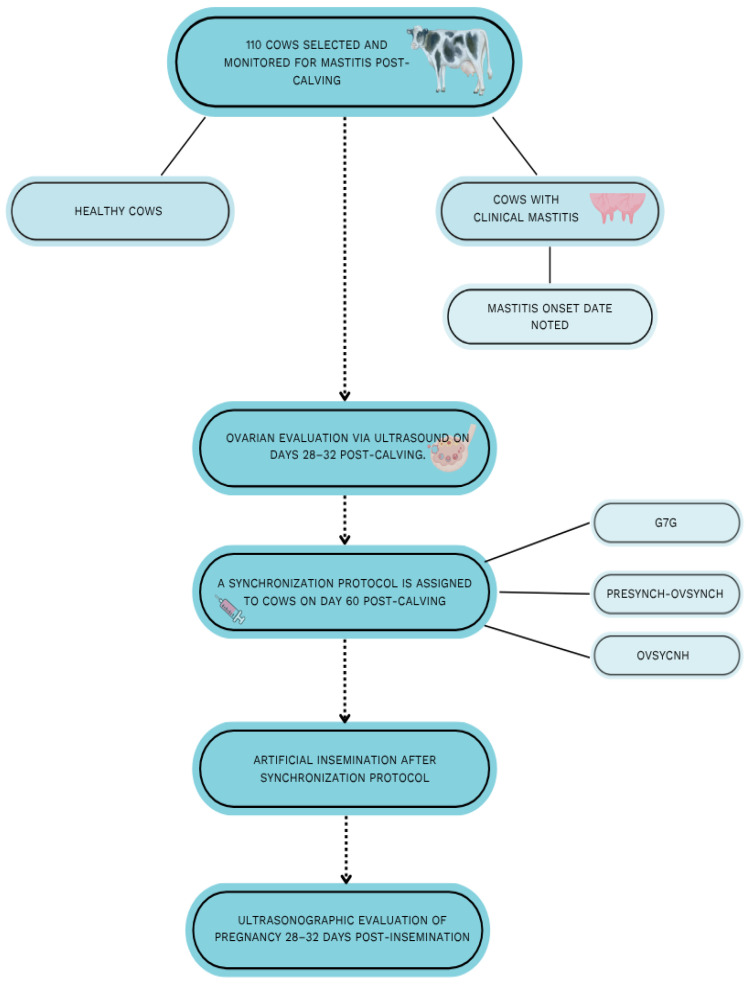
A visual plan of the experimental methodology.

The animals were subsequently divided into two groups: Group 1, cows that remained healthy from 0 to 30 days pp (*n* = 53), and Group 2, cows that developed clinical mastitis within the same period (*n* = 57) (Figure 2). In the group of cows that developed clinical mastitis, the date of mastitis onset was recorded. Mastitis cases were categorised further into two intervals based on the day of onset: <30 days postpartum (*n* = 37) and >30 days postpartum (*n* = 20). Throughout this study, all cows were kept under the same conditions and fed the same diet. Regardless of whether udder infection occurred or not, all cows underwent an ultrasonographic evaluation of their ovaries and the structures within them on days 28–32 postpartum. Visual diagnostics were performed during rectal examination using the “KAIXIN RKU10” ultrasound device (Xuzhou Kaixin Electronic Instrument Co., Ltd., Xuzhou, China, 2013) with a 6.5 MHz frequency. The area of the left and right ovaries, as well as the number and size of the structures, was recorded. Ovarian area was calculated using the “measure” function by taking the greatest visible width and length. Similar actions were performed to record the follicles and the CLs using the “freeze” parameter. The ovarian area was calculated using the mathematical formula ovarian area = ovary length × ovary width.

All synchronisation protocols were initiated at exactly 60 days in milk (DIM) for each cow, regardless of group assignment. The first injection or treatment in each protocol (OvSynch, G7G, or Presynch) was administered at this fixed point (60 DIM). As a result, the timing of artificial insemination (AI) depended on the length of the specific protocol: AI was performed at the end of each protocol sequence according to its standard schedule. Consequently, the actual DIM at AI varied slightly among the protocols due to their different durations.

Cows were randomly assigned to the three protocols upon reaching 60 DIM. This approach reflects typical farm practice, where synchronisation programmes are started at a targeted DIM, and insemination occurs as prescribed by the protocol length. The random assignment minimised bias related to health status, milk yield, or other factors.

The cows were assigned to the protocols in a randomised manner. In Group 1 (*n* = 53), 18 cows received the Presynch protocol, 17 were treated with OvSynch, and 18 were treated with G7G, while in Group 2 (*n* = 57), 19 cows were assigned to each of the three protocols. The medications used for the injections were “Ovarelin” (50 μg/mL of gonadorelin diacetate tetrahydrate, Ceva Santé Animale, Libourne, France) and “Oestrophan” (250 μg of cloprostenol, Bioveta, a.s., Ivanovice na Hané, Czech Republic). A detailed model of the synchronisation protocols is presented in Figure 1 and Figure 2. Throughout the synchronisation protocol, injections were administered at 8:00 AM. After completing the selected synchronisation protocol, the cows were inseminated with cryopreserved semen during artificial insemination. Twenty-eight to thirty days after insemination, early pregnancy status was evaluated using ultrasound. A simplified visual plan of the study methodology is presented in Figure 2.

The impact of mastitis on the reproductive cycle and ovarian function was assessed through ultrasonographic examination of the ovaries (measuring their area and structures) and by evaluating reproductive performance through conception rates and the response to the synchronisation protocols. Hormonal profiles and detailed oestrous cycle stages were not directly monitored; rather, reproductive function was inferred from ovarian morphology and fertility outcomes.

### 2.4. Statistical Analysis

The collected data were recorded and processed using Microsoft Excel 2016 (Microsoft Corporation, Redmond, WA, USA). The statistical analyses were conducted using IBM SPSS Statistics 26.0 (IBM Corp., Armonk, NY, USA). Descriptive statistics, including arithmetic means and standard deviations, were calculated. Comparisons between independent groups (e.g., cows with and without mastitis) were performed using Student’s *t*-test. Dependent sample comparisons (e.g., the left and right ovaries within the same cow) were analysed using paired *t*-tests. The analysis of fertilisation efficiency was performed using a two-way ANOVA. A significance level of *p* < 0.05 was considered statistically significant. Graphical representations were created using Microsoft Excel 2016 and Microsoft Word 2016.

## 3. Results

### 3.1. Ovarian Area and Ovarian Derivatives Postpartum in Cows with Mastitis and with Healthy Ovaries

#### 3.1.1. The Size of the Ovaries in Affected and Healthy Cows

**Figure 3:**
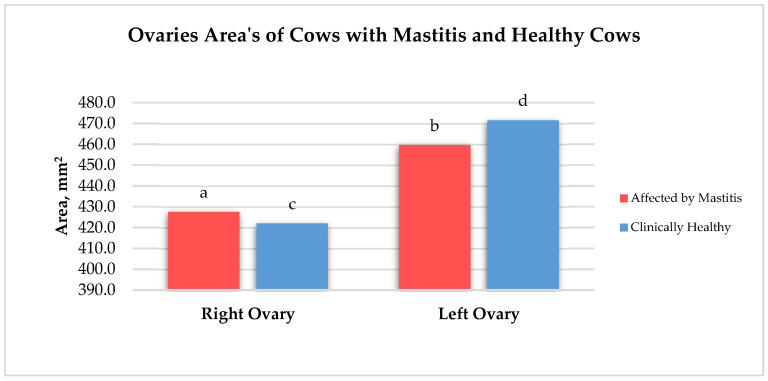
Areas of ovaries of healthy cows and affected by mastitis, mm^2^. a:b (*p* < 0.05); a:c (*p* > 0.05); c:d (*p* < 0.05); c:b (*p* > 0.05); a:d (*p* > 0.05).

The mean areas of the left and right ovaries for both groups are presented in Figure 3. In the cows affected by mastitis, the left ovary was 6.97% larger than the right ovary. In healthy cows, the left ovary was 10.51% larger than the right ovary (*p* < 0.05) in the same group of cows. The left ovary in the healthy cows was 2.53% larger (*p* > 0.05) compared to that in the group of cows with mastitis in the left ovary. The right ovary in cows with mastitis was 1.3% larger (*p* > 0.05) than the right ovary in healthy cows.

#### 3.1.2. The Number of Ovarian Derivatives in Cows Affected by Mastitis and Healthy Cows

**Figure 4:**
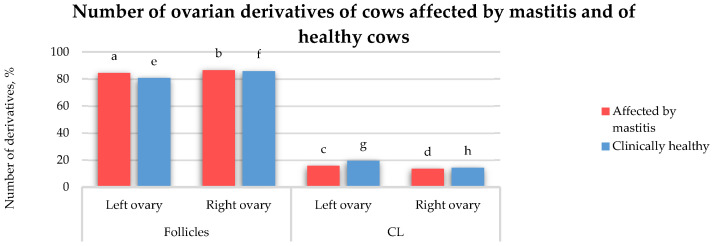
Number of ovarian derivatives in cows affected by mastitis and healthy cows, %. a:b (*p* > 0.05), a:c (*p* < 0.05), a:e (*p* > 0.05), b:d (*p* < 0.05), b:f (*p* > 0.05), c:d (*p* > 0.05), c:g (*p* > 0.05), d:h (*p* > 0.05), e:f (*p* > 0.05), e:g (*p* < 0.05), f:h (*p* < 0.05), g:h (*p* > 0.05).

The analysis of the ovarian structures (Figure 4) in clinically healthy cows revealed that the number of follicles in the left ovary was 5.06% lower (*p* > 0.05) compared to that in the right ovary. In addition, the number of CLs in the left ovary was 5.06% higher (*p* > 0.05) than that in the right ovary. In the ovaries of the cows affected by mastitis, the number of follicles in the left ovary was 2.07% lower (*p* > 0.05) compared to that in the right ovary. Conversely, the number of CLs in the left ovary was 2.07% higher (*p* > 0.05) than that in the right ovary. In the cows with mastitis, the left ovary contained 3.72% more follicles (*p* > 0.05) than the left ovary in healthy cows. Similarly, the right ovary in the cows with mastitis had 0.73% more follicles (*p* > 0.05) than the right ovary in healthy cows. In the left ovary of the healthy cows, the number of CLs was 3.72% higher (*p* > 0.05) than that in the left ovary of the cows affected by mastitis. In contrast, in the right ovary, cows with mastitis exhibited 0.73% fewer CLs (*p* > 0.05) compared to those in healthy cows. In the cows with mastitis, the left ovary contained 68.74% more follicles than CLs (*p* < 0.05), while the right ovary showed a 72.88% greater number of follicles than CLs (*p* < 0.05). In healthy cows, the left ovary contained 61.3% more follicles (*p* < 0.05) than CLs, while the right ovary had 71.42% more follicles (*p* > 0.05) than CLs.

#### 3.1.3. The Size of the Ovarian Structures

**Figure 5:**
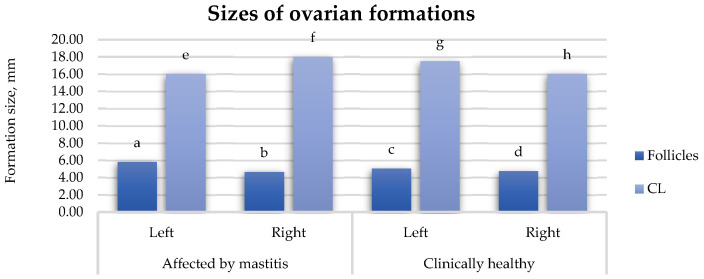
Sizes of ovarian derivatives in cows affected by mastitis and healthy cows, mm. a:b (*p* > 0.05); a:c (*p* > 0.05); b:d (*p* > 0.05); c:d (*p* > 0.05); e:f (*p* < 0.05); e:g (*p* > 0.05); f:h (*p* < 0.05); g:h (*p* > 0.05); cows, %. a:b (*p* > 0.05); a:c (*p* < 0.05); a:e (*p* > 0.05); b:d (*p* < 0.05); b:f (*p* > 0.05); c:d (*p* > 0.05); c:g (*p* > 0.05); d:h (*p* > 0.05); e:f (*p* > 0.05); e:g (*p* < 0.05); f:h (*p* < 0.05); g:h (*p* > 0.05).

In the group of cows affected by mastitis, the follicles in the left ovary were 19.93% larger than those in the right ovary (*p* > 0.05) (Figure 5). Alternatively, the CLs in the right ovary were 11.11% larger than those in the left ovary (*p* < 0.05). In the group of healthy cows, the follicles in the left ovary were 6.34% larger than those in the right ovary (*p* > 0.05). The CLs in the left ovary were 8.57% larger compared to those the right ovary (*p* > 0.05). Comparing the groups, the follicles in the left ovaries in the cows with mastitis were 13.23% larger than those in the left ovaries of healthy cows (*p* > 0.05). Follicles in the right ovary of the cows with mastitis were 1.48% larger than those in healthy cows (*p* > 0.05). Regarding CLs, in the left ovary, they were 8.57% smaller in the cows affected by mastitis compared to the healthy group (*p* > 0.05). However, in the right ovary, the CLs were 11.11% larger in cows with mastitis than those in healthy cows (*p* < 0.05).

### 3.2. The Effect of the Synchronisation Protocol on Fertilisation Efficiency in Cows with Mastitis and Healthy Cows

#### 3.2.1. Fertilisation Efficiency Based on the Number of Inseminations

**Figure 6:**
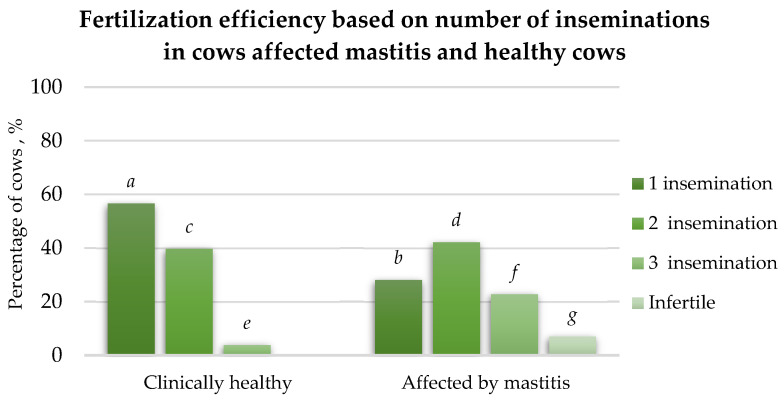
The dependence of fertilisation rates on the number of inseminations in cows affected by mastitis and healthy cows, %. a:b (*p* < 0.05); a:c (*p* < 0.05); b:d (*p* < 0.05); c:d (*p* > 0.05); c:e (*p* < 0.05); d:f (*p* < 0.05); e:f (*p* < 0.05); f:g (*p* < 0.05).

After the first insemination, the fertilisation efficiency was 28.53% higher (*p* < 0.05) in the healthy cow group compared to that in the mastitis-affected group of cows (Figure 6). After the second insemination, a 2.49% higher fertilisation rate (*p* > 0.05) was observed in the mastitis-affected cows compared to that in healthy cows. However, the third insemination was 19.03% more effective (*p* < 0.05) in the mastitis-affected cow group than in healthy cow group. In the healthy group of cows, the fertilisation efficiency after the first insemination was 16.98% higher (*p* < 0.05) compared to that after the second insemination. The second insemination in healthy cows was 35.84% more effective (*p* < 0.05) than the third insemination. In the mastitis-affected group of cows, the fertilisation efficiency was 14.04% higher (*p* < 0.05) after the second insemination than the first. Additionally, 19.3% (*p* < 0.05) of the cows required a third insemination after failing to conceive after the second insemination. After the third insemination, 7.01% (*p* < 0.05) of the mastitis-affected cows remained infertile.

#### 3.2.2. The Effect of Different Synchronisation Protocols on First Insemination Fertilisation

**Figure 7:**
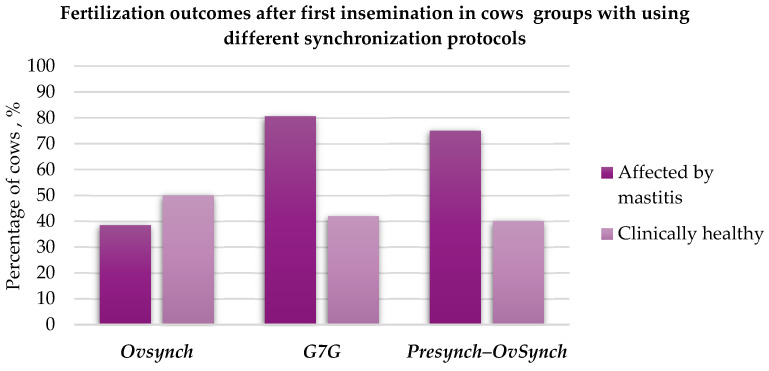
First insemination fertilisation rates in cows with mastitis and healthy cows using different synchronisation protocols, %.

In the group of cows with mastitis, the OvSynch protocol resulted in a 42.11% (*p* < 0.05) decrease in fertilisation efficiency in comparison to that under the G7 protocol and a 36.55% (*p* < 0.05) decrease in fertilisation efficiency in comparison to that under the Presynch protocol. In the healthy cow group, the OvSynch protocol resulted in an 8.05% lower fertilisation efficiency compared to that under the G7G protocol and a 10% lower efficiency compared to that under the Presynch protocol (Figure 7).

### 3.3. Fertilisation Efficiency in Cows with Mastitis and Healthy Cows Based on the Onset of Mastitis

#### 3.3.1. The Onset of Mastitis After Parturition

**Figure 8:**
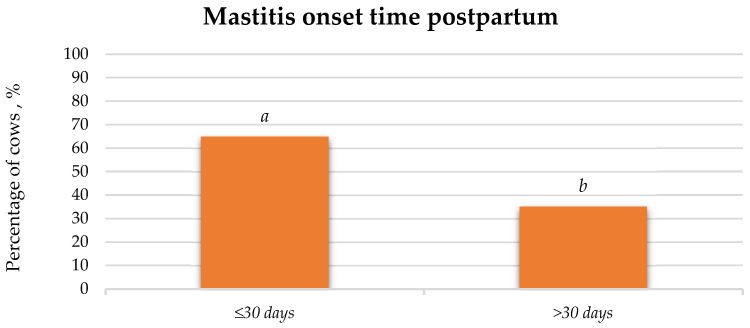
Incidence of mastitis onset after parturition, %. a:b (*p* < 0.05).

During this study, it was found that mastitis, regardless of its type, occurred 29.82% more frequently (*p* < 0.05) within 30 days after parturition (Figure 8).

#### 3.3.2. Fertilisation Efficiency Depending on the Onset of Mastitis

**Figure 9:**
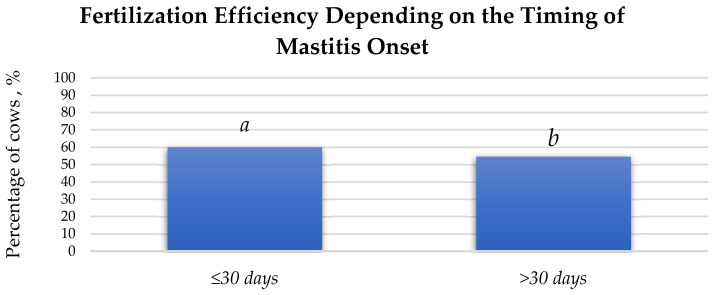
The dependence of fertilisation efficiency after first insemination on the timing of mastitis onset %. a:b (*p* > 0.05).

A higher fertilisation efficiency was observed in the group of cows that developed mastitis within 30 days after parturition (Figure 9). After the first insemination, cows that developed mastitis within 30 days postpartum had a 5.45% higher fertilisation rate (*p* > 0.05) than that in cows that developed mastitis 30 or more days after parturition.

## 4. Discussion

Variation in ovarian size is influenced by multiple factors, including the stage of the reproductive cycle, differential ovarian activity, the size and condition of the uterine horns, and reproductive system pathologies. In most animal species, ovarian function is asymmetric, with one ovary exhibiting greater activity than the other [24]. Karamishabankareh et al. [19] reported that this asymmetry in ovarian activity is associated with differences in the timing of ovulation and oocyte development between the two ovaries. In our study, the left ovary was observed to be larger and to exhibit more structural formations in both the affected and healthy cows than the right ovary. However, some studies have suggested that the right ovary has a higher ovulation rate and produces a greater number of ovarian structures [20,21]. Currently, no scientific consensus exists regarding the functional dominance or size superiority of the right ovary. It may be hypothesised that ovarian predominance is an individual characteristic in each animal.

A recurrent finding in the scientific literature is that mastitis adversely affects the reproductive performance of cows. The onset of mastitis at the beginning of lactation is associated with the physiological stress of calving and a rapid increase in milk secretion and later in the lactation period is associated with physical damage to the udder [22]. In our study, udder infections were more frequent fewer than 30 days after calving. In a study by Elmaghraby et al. [25], the majority of the cows with clinical mastitis were recorded in the first month of lactation after calving, and the service period of the affected cows was extended by 43 days. The authors also mentioned that some of the subjects had mastitis at least once in the first 3 months postpartum. The adverse effects of clinical mastitis have been attributed to the overproduction of bacterial toxins in the body that stimulate prostaglandin secretion. High concentrations of PGF2α have been shown to lead to luteolysis of the yolk sac, decreased progesterone levels, prolonged follicular maturation, and embryonic death [26]. Ahmadzadeh et al. [27] reported that the occurrence of mastitis more than 56 days after calving required more artificial insemination manipulations compared with those in cows that developed the disease earlier. This study revealed that a higher proportion of cows with mastitis than healthy cows was not pregnant. Conversely, contrasting results were obtained in a study by Boujenane et al. [28], in which the cows were grouped according to disease period. In one of the groups, cows with disease < 60 days postpartum were not affected by disease during this period. In contrast, the present study documented a reduced incidence of disease in cows 30 days or more after calving. We propose that alterations in the ovarian structures at 28 DIM may reflect underlying systemic conditions—such as inflammation, immune dysregulation, or metabolic stress—that predispose cows to mastitis later in lactation. In this context, the ovarian parameters function not as outcomes of mastitis but as early physiological indicators of a cow’s overall health status and disease susceptibility. This concept is supported by previous research. In a field trial by Hockett et al. [29], cows that developed mastitis between 15 and 28 DIM exhibited delayed resumption of ovarian cyclicity, reduced ovulation rates by day 28, and an increased incidence of premature luteolysis—especially in cases caused by Gram-negative bacteria or culture-negative pathogens. These disruptions occurred prior to or in parallel with the clinical manifestation of mastitis, suggesting that early ovarian abnormalities may be linked to a systemic environment conducive to disease development. Moreover, experimental studies have demonstrated that acute udder infections can suppress luteinising hormone (LH)’s pulsatility and reduce estradiol-17β production, thereby impairing oestrous expression and follicular development [29]. Rahman et al. [30] further showed that cows with chronic mastitis exhibited impaired folliculogenesis, including a disrupted transition from the primary to secondary follicles and reduced development of dominant follicles, reinforcing the impact of systemic inflammation on ovarian physiology. Therefore, our interpretation is that the differences in ovarian size and morphology observed at 28 DIM may serve as predictive markers of cows at increased risk of subsequent mastitis, rather than being a consequence of mastitis itself. We have clarified this interpretation in the revised version of this manuscript to reflect the directionality and implications of our findings better.

In the study by Lavon et al. [31], the impact of mastitis on reproductive processes and its association with prolonged pre-ovulatory intervals were analysed. In 70% of the cows affected by mastitis studied, their reproductive performance remained unchanged, but in about 30% of the cows, ovulation was delayed. The oestrus interval increased from 30 to 60 h, reducing the probability of successful insemination in inseminated cows. In our study, the calving rate was observed to be almost 30% higher in healthy cows than in sick cows. Furthermore, the second insemination was found to be more successful in the cows with mastitis than in the healthy group.

The prevailing consensus in the extant literature is that mastitis, irrespective of its specific type, exerts a deleterious effect on reproductive performance. A study by Smulski et al. [32] found that cows afflicted with mastitis exhibited a protracted service period, a high insemination index, and a reduced conception rate and that the frequency of insemination was marginally lower in healthy cows that healed successfully than that in those that had to undergo protracted treatment. The detrimental effect of mastitis on fertility is hypothesised to be attributable to the 7–10 days of treatment and the ongoing inflammatory process, either due to the low efficacy of antimicrobial therapy or to a lack of supportive care.

A study by Cheong et al. [33] investigated the impact of inflammatory responses in the uterus and the whole body at the time of parturition on the development and ovulatory function of the first dominant follicle in dairy cows in the postpartum period. Their findings indicated that cows with a high initial uterine immune response, as indicated by elevated polymorphonuclear leukocyte counts and reduced uterine pH levels, exhibited an increased tendency to ovulate. This suggests that a prompt local defence mechanism may facilitate ovarian recovery. In contrast, cows with higher systemic inflammation, as indicated by elevated haptoglobin levels, exhibited a reduced probability of ovulation, despite similar bacterial contamination and circulating endotoxin concentrations [34,35]. Furthermore, non-ovulatory cows exhibited elevated endotoxin levels in the follicular fluid and reduced follicular growth rates. It is evident that regardless of the underlying disease, inflammatory mediators have a detrimental effect on reproductive performance. Regardless of the underlying disease, inflammatory mediators appear to negatively affect reproductive performance, and based on the findings presented, it is reasonable to assume that similar disruptions to ovarian function may occur in cases of mastitis. These results highlight that while a localised uterine inflammatory reaction may have a protective role, prolonged or excessive systemic inflammation in the early postpartum period can impair ovarian function, underscoring the influence of the whole body’s inflammatory status on reproductive efficiency in dairy cows.

Inflammatory processes within the body, such as those initiated by endotoxin exposure, can lead to a systemic response marked by an elevated body temperature and a reduction in serum paraoxonase activity [36], an enzyme associated with antioxidant defence. This systemic inflammation has been shown to adversely influence reproductive hormones, particularly by suppressing the pulsatile release of luteinising hormone (LH) from the anterior pituitary gland, a key regulator of follicular growth and ovulation. A diminished frequency of LH pulses is a well-recognised feature in cows that fail to ovulate [37]. Evidence also suggests that cows which resume ovarian cyclicity earlier in the postpartum period—specifically those ovulating within 44 days—tend to have a more favourable inflammatory profile. These animals typically exhibit lower concentrations of haptoglobin prior to calving and higher levels of serum paraoxonase and albumin after parturition compared with those in cows that remain anovulatory [38]. Such findings highlight a potential link between systemic inflammation and compromised ovarian function, underscoring the sensitivity of the hypothalamic–pituitary–ovarian axis to inflammatory mediators. In the context of mastitis—a prevalent inflammatory condition in dairy herds—the implications for reproductive performance are particularly concerning. The inflammatory cascade triggered by mammary gland infection may not only impair general health and metabolic stability but also exert indirect effects on ovarian morphology and follicular development, ultimately disrupting normal reproductive rhythms.

The extant literature supports the hypothesis that there is a link between chronic mastitis and disrupted ovarian function in dairy cows. Both clinical and subclinical intramammary infections have been demonstrated to have a detrimental effect on fertility, likely via alterations in ovarian and follicular dynamics. As reported by Rahman et al. [30], the progression from the primary to secondary follicles was found to be impaired and the development of dominant follicles was reduced in cows afflicted with severe udder infections. The present study provides the first histological evidence of mastitis-associated ovarian changes, including reduced vascularisation and stromal fibrosis. These vascular deficits have the potential to impede the process of follicular maturation, a finding that is consistent with in vitro observations, where vascular endothelial growth factor has been shown to promote follicular transition. Furthermore, the reduced expression of oocyte-specific factors, such as human growth and differentiation factor-9, suggests a direct impact on folliculogenesis. In summarising the findings of this study, it is evident that chronic mastitis instigates structural ovarian changes, including vascular reduction, fibrosis, and altered regulatory signalling, which collectively contribute to a decline in fertility.

In the present study, three synchronisation protocols were utilised: OvSynch, G7G, and Presynch. OvSynch is the most prevalent synchronisation protocol in dairy herds; however, its efficacy is contingent on the size of the follicles in the ovaries at the time of the initial GnRH injection [38,39]. The present study indicated that following the first insemination, the OvSynch synchronisation protocol produced the highest number of cows that calved, irrespective of the cow’s condition, whether she was sick or healthy. A similar result was obtained in a study by Hassan et al. [40] which showed a calving rate of 43% after the first insemination with the OvSynch protocol and a rate of 31% with prostaglandin therapy. Lavon et al. [18] investigated the probability of insemination in cows with subclinical mastitis and healthy cows treated with the OvSynch protocol, finding that the OvSynch protocol resulted in a marginal difference in the insemination rates of the affected cows compared to those in healthy cows. The protocol’s application window extends from 5 to 12 days of the reproductive cycle, and it is hypothesised that this adjustment to the reproductive cycle is likely to result in positive outcomes.

A substantial number of scientific papers suggest that presynchronisation with prostaglandins 14 days before the OvSynch protocol results in a higher probability of fertilisation, a modification known as the Presynch protocol [41,42]. Since presynchronisation with one PGF may not be successful in cows with a functioning CL, it is recommended to presynchronise with two PGF injections 14 days apart [43]. This hypothesis is substantiated further by numerous authors of the scientific papers referenced in the retrospective study by Colazo et al. [41] which analysed synchronisation protocols in beef and dairy cattle. However, Borchardt et al. [42] posit that presynchronisation results in a reduction in the probability of insemination. The enhanced fertilisation efficiency evident following the initial insemination of cows administered the Presynch and G7G synchronisation protocols, relative to that under Ovsynch alone, can be ascribed to the integration of a presynchronisation phase. This phase ensures that cows are synchronised with the optimal stage of the oestrous cycle prior to the initiation of Ovsynch. The G7G protocol involves the administration of PGF2α to induce luteolysis of the functional corpora lutea (CL), followed by GnRH two days later to stimulate ovulation [2]. This sequence has been demonstrated to promote the formation of a new CL, thereby enhancing the response to PGF2α and the second GnRH injections in the Ovsynch protocol, thus leading to an improvement in the synchronisation efficiency [3]. Nonetheless, research has indicated that dairy cows initiated on the Ovsynch protocol during late dioestrus exhibit diminished responsiveness to the initial GnRH injection. This has been demonstrated to compromise synchronisation and result in a reduction in pregnancy rates. It is possible that cows may fail to ovulate in response to the first GnRH injection and undergo spontaneous luteolysis before the scheduled PGF2α injection, thereby triggering an early LH surge and ovulation prior to timed artificial insemination and consequently reducing the likelihood of conception [3]. In support of this, Dirandeh et al. [44] demonstrated that presynchronisation protocols such as Double Ovsynch (DO) and G7G significantly increased the ovulatory response to the first GnRH injection in Ovsynch. Furthermore, an increased ovulation rate was observed in the DO (74.0%) and G7G (76.0%) groups in comparison to that in the Ovsynch-only group (50.0%). In addition, pregnancy through AI at 32 days post-insemination was found to be significantly higher in the G7G (32.7%) and DO (31.1%) groups than in the Ovsynch group (19.7%).

Mastitis is a condition that has been widely acknowledged not only for its substantial economic outcomes but also for its profound impact on the health and well-being of the affected animal. According to research carried out by Hultgren and associates in Sweden, the incidence of clinical mastitis rate per lactation was reported at 14 percent, equating to an annual occurrence of approximately 0.11 cases per cow [45]. In this regard, growing scientific interest has been directed towards the ovarian reserve as a potential indicator of fertility, particularly in cattle with prolonged productive lifespans. The association between reproductive capacity and the number of antral follicles—measured via the antral follicle count (AFC)—has garnered considerable attention. Favoreto et al. [46] reported that cows exhibiting higher AFCs also possessed a greater number of preantral follicles, thereby reinforcing the relationship between follicular populations and overall ovarian function. Building on the findings of different studies, Alvarez et al. [47] conducted a study involving Nellore cows managed under extensive grazing systems and confirmed a positive correlation between average ovarian size and AFC. Combining these findings, it can be concluded that monitoring ovarian parameters such as the AFC and ovarian size may offer a valuable, non-invasive approach to assessing the fertility potential in dairy cattle, particularly those at risk of health-related reproductive declines such as mastitis. The ovarian structures play a critical role in successful reproduction; further research is warranted to deepen our understanding of their function and potential as predictive markers of fertility.

SCC serves as a reliable biomarker for detecting subclinical mastitis [48]. Research has demonstrated a correlation between an elevated SCC at the time of insemination and impaired conception rates, whilst lower values have been shown to be associated with enhanced fertility outcomes [49]. McDougall et al. [50]’s observations revealed a decline in milk yield during oestrus in goats subjected to increased SCC levels under various synchronisation protocols. Subsequent research by Lavon et al. [18] demonstrated that the Ovsynch protocol led to the restoration of pregnancy rates in mastitic cows to levels comparable with those observed in unaffected animals. In support of this finding, the results of Saat et al. [51] indicated that amongst the range of oestrus synchronisation methods examined in Simmental cows, the Ovsynch protocol resulted in the lowest increase in the bulk tank milk somatic cell count, exhibiting no significant difference when compared to that in the untreated control groups. Their study, which included a comparison with progesterone-releasing intravaginal devices and double-dose PGF2α treatments, suggested that spontaneous oestrus and Ovsynch are preferable options for synchronisation due to their lesser impact on udder health. In the present study, the G7G and Presynch protocols were found to enhance the insemination performance in the cows afflicted with the disease in comparison to that in the OvSynch group. The practicality of these protocols is evidenced by the fact that the majority of the injections are administered at weekly intervals [42]. Dirandeh et al. [52] reported in their study that the G7G protocol resulted in a higher probability of ovulation, smaller ovulatory follicles, and a higher calving index compared with these values under the OvSynch synchronisation programme. A limitation of this study is that only early pregnancy was confirmed using ultrasonography at 28–30 days post-insemination, and the cows were not monitored until calving. Therefore, potential pregnancy losses or calving disorders beyond early gestation were not assessed. Additionally, although cows with obvious health problems other than clinical mastitis were excluded, it is possible that subclinical disorders or metabolic imbalances may have affected the reproductive outcomes. Future research should include longer-term monitoring and broader health screening to fully understand the impact of mastitis and other confounding factors on fertility.

## 5. Conclusions

This study demonstrates that clinical mastitis in dairy cows leads to measurable changes in ovarian morphometry, as evidenced by differences in the area, number, and size of follicles and corpora lutea compared to those in healthy cows. These morphometric alterations were associated with impaired reproductive outcomes, including a reduced fertilisation efficiency following synchronisation and artificial insemination. Additionally, the effectiveness of the oestrous synchronisation protocols varied depending on the health status of the cows. The G7G protocol was found to be more effective in improving the conception rates in cows with clinical mastitis, while the OvSynch protocol showed better outcomes in healthy cows. The timing of mastitis onset also played a role, with early postpartum cases exhibiting more favourable fertilisation rates after the first insemination. Overall, our findings highlight the importance of adapting reproductive management strategies, including the choice of synchronisation protocol, in dairy cows affected by mastitis. Ultrasonographic assessment of ovarian parameters provides valuable insights for optimising fertility protocols and improving reproductive performance in herds where clinical mastitis is a concern.

Future research should aim to elucidate the pathogen-specific mechanisms and inflammatory pathways linking mastitis to ovarian dysfunction, with the goal of optimising fertility protocols in dairy herds.

It is recommended that future scientific research aim to identify the specific pathogens responsible for mastitis and elucidate the inflammatory pathways linking mastitis to ovarian dysfunction. The ultimate goal of such research would be to optimise the fertility protocols in dairy herds. Furthermore, grouping cows into narrower categories based on days in milk (DIM) at the onset of mastitis could provide a more specific and biologically appropriate understanding of how the timing of infection influences reproductive outcomes. The identification of critical periods of vulnerability would be facilitated by such stratification, as would the implementation of more targeted reproductive management and treatment strategies.

## Figures and Tables

**Table 1:** Composition of dry and total mixed feed rations.

Dry Matter			Total Mixed Ratio		
Parameters	Units	Quantity	Parameters	Units	Quantity
Dry matter	%	48.0	Corn silage	%	24.0
Acid detergent fibre	%	20.0	Grass hay	%	5.0
Non-fibre carbohydrates	%	39.0	Grass silage	%	16.0
Neutral detergent fibre	%	28.0	Grain concentrate slurry	%	50.0
Crude protein	%	16.0	Mineral mixture	%	5.0

## Data Availability

The data in this work can be obtained upon request from the corresponding author.

## Abbreviations

The following abbreviations are used in this manuscript:

DIM	Days in milk
NEB	Negative energy balance
DMI	Dry matter intake
BHB	Beta-hydroxybutyrate
LPS	Lipopolysaccharide
AI	Artificial insemination
CL	Corpus luteum
PGF2α	Prostaglandin f2α
GnRH	Gonadotropin-releasing hormone
PO	Presynch–Ovsynch
pp	Postpartum
LH	Luteinising hormone
AFC	Antral follicle count
PGF	Prostaglandin f_2α_
SCC	Somatic cell count
